# Advanced QT interval analysis in long-term electrocardiography using shape-based clustering and template matching: A novel approach for Holter monitoring

**DOI:** 10.1016/j.heliyon.2025.e42662

**Published:** 2025-02-13

**Authors:** Kaoru Hatano, Tomohiro Takata, Mineki Takechi, Akane Wada, Hirohisa Taniguchi, Yuichi Tamura

**Affiliations:** a Cardio Intelligence Inc, 1-25-5 Higashiazabu, Minato-ku, Tokyo, 106-0044, Japan; b Advanced Graduate Program for Future Medicine and Health Care, Tohoku University, 2-1 Seiryo-cho, Aoba-ku Sendai, Miyagi, 980-8574, Japan; c Department of Cardiology, International University of Health and Welfare Mita Hospital, 1-4-3 Mita, Minato-ku, Tokyo, 108-8329, Japan

**Keywords:** QT interval, QT prolongation, K-shape clustering, Dynamic time warping under limited warping path length, Template matching, Electrocardiogram

## Abstract

**Introduction:**

The QT interval, a critical parameter in electrocardiography (ECG), is challenging to analyze accurately because of variations in T-wave morphology, especially in long-term ECG recordings, such as Holter monitoring. Current methods, which are often manual or semi-automated, lack consistency and efficiency, underscoring the need for a more reliable and automated approach.

**Methods:**

We developed a novel QT interval analysis algorithm that integrates K-shape clustering with Dynamic Time Warping under Limited Warping Path Length for template matching. This method automatically categorizes ECG waveforms into clusters based on shape similarity, and then selects representative template waveforms for each cluster. This approach aimed to standardize and automate the process of identifying and analyzing QT intervals across varying waveform patterns.

**Results:**

Validation of the algorithm using the QT Database, which encompasses a broad spectrum of ECG waveform patterns, confirmed its ability to accurately identify and analyze the QT interval across diverse T-wave morphologies, in close agreement with expert human analysis. The reliability of the algorithm was quantified using the Intraclass Correlation Coefficient. For the QT interval, the Intraclass Correlation Coefficient (2,1) was 0.9 [95 % CI: 0.89, 0.91], and for the QTcB interval, it was 0.82 [95 % CI: 0.80, 0.84], both reflecting high reliability. The reliability of the algorithm was also evaluated by comparing it with DSC5500 from Nihon Kohden analysis program for long-time ECGs, a commercial software program that uses the tangential method. For the QT interval calculated by the developed algorithm, the Intraclass Correlation Coefficient (2,1) was 0.95 [95 % CI: 0.92, 0.97], and for the QT interval calculated by the DSC 5500, it was 0.70 [95 % CI: 0.54, 0.81]. For all waves, the mean and standard deviation of the difference between the algorithm and the expert analysis was 0.5 (±16), and that of the difference between the DSC5500 and the expert analysis was −16.4 (±42.8). For biphasic T-wave, the mean and standard deviation of the difference between the algorithm and the expert analysis was −0.3 (±15.9), and that of the difference between the DSC5500 and the expert analysis was −79.3 (±60.6). The results showed that the developed algorithm performed better than the DSC5500 for all shapes, especially for biphasic T-wave. The algorithm also demonstrated enhanced time efficiency and streamlined QT interval analysis in clinical settings.

**Discussion:**

The new algorithm enhanced automated QT interval analysis, especially for long-term ECG recordings. Its effectiveness in processing different T-wave morphologies with accuracy and efficiency makes it a promising tool for clinical use and improving QT interval analysis in patient care and diagnostics.

## Highlights

• Manual template generation is a challenge for QT analysis by template matching.
• Template generation was automated applying the K-shape clustering.
• New QT analysis method can reduce the burden of manual analysis of Holter ECGs.

## Graphical abstract

**Figure:**
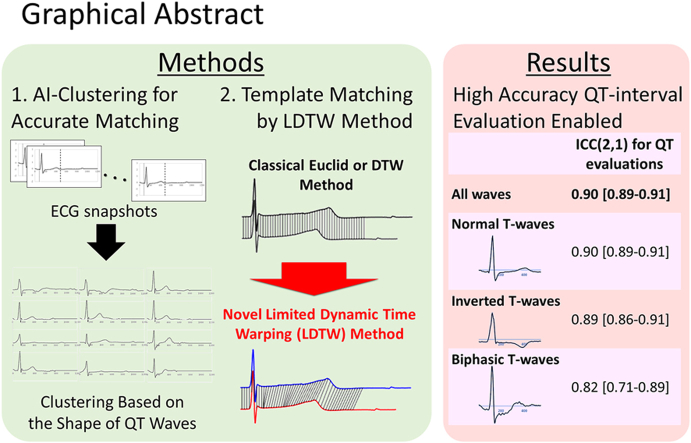

## 1 Introduction

Drug-induced QT prolongation is one of the factors that can cause life-threatening arrhythmias, such as Torsade de Pointes[[1], [2], [3], [4]]. QT interval measurements are typically performed using a 12-lead electrocardiogram for a period of 5–10 s. However, daily variability in QT intervals is usually observed [5]. For instance, QT intervals frequently fluctuate in response to varying blood drug concentrations [6,7], complicating accurate assessment of drug-induced QT prolongation using only short-duration measurements. Consequently, methodologies employing extended-duration electrocardiography (ECG) have been proposed and implemented for comprehensive QT interval measurements and analysis [8,9].

The topic of automatic QT intervals analysis of ECG has been widely studied. One can find in the literature very different approaches based on Mathematical models [10], matched filters [11], ECG slope criteria [12], low-pass differentiation [13], the wavelet transform [14], hidden Markov models [15]. In recent years, QT interval analysis using neural networks has been actively studied, with reports demonstrating high accuracy [[16], [17], [18]]. However, when using neural networks, the lack of a validation process for measurements in clinical settings presents a significant barrier to clinical application. To address this issue, some methods have been proposed to enhance the explainability of AI-generated TdP (Torsades de Pointes) risk assessments by visualizing the degree of QT prolongation through color enhancement [19,20]. On the other hand, there is currently no standardized, fully automated method established for QT interval analysis in long-term electrocardiography (such as Holter ECGs) with limited electrical leads. For the analysis of QT intervals over extended periods, the capability to process large datasets efficiently and consistently is essential. As traditional methods alone are insufficient to meet these demands, technological advancements are important. Therefore, it is recommended that the QT interval analysis be either completely manual or manually assisted by computers[[21], [22], [23]]. The rationale for this recommendation is that conventional automated analysis methods have low reliability, especially in cases where the morphology of the T-wave changes [24,25]. Furthermore, some drugs can alter T-wave morphology when administered [26]. In contrast, the ECG waveform of a long-term ECG contains approximately 100,000 beats in a 24-h period, making it difficult to perform every analysis manually.

Given these issues, it has traditionally been difficult to perform real-time evaluation of drug-induced QT prolongation in accordance with pharmacokinetic variations. One key to achieving continuous long-term assessment of QT duration is to establish an algorithm capable of accommodating morphological changes in T waves. The template-matching method is a promising approach for addressing the issue of accuracy in analyzing T-wave morphological changes in long-term ECG QT analysis[[27], [28], [29]]. However, this method often necessitates manual creation of templates, which requires considerable human resources. To address this problem, attempts have been made to automate template creation. Nevertheless, the disparity in the identification of the T-wave ends between experts and the proposed automated method presents an issue in terms of accuracy [30].

In this study, aiming for a semi-automated and highly accurate QT analysis, we developed a model that improves the traditional template-matching method. Electrocardiogram waveforms were segmented beat by beat and then classified into similar waveforms using the K-shaped method [31]. This approach allows for the semi-automated creation of templates by automatically extracting template waveforms for each similar-shape classification.

Furthermore, for the template matching method in QT analysis, we adopted a variant of the Dynamic Time Warping (DTW) method, known as DTW under Limited Warping Path Length (LDTW) method [32]. This method limits the search range during the alignment of electrocardiogram waveforms, thereby enhancing the matching precision. This study aimed to establish these methods and evaluate their external validity.

## 2 Materials and Methods

### 2.1 Preprocessing of ECG data

Heartbeat waveform extraction: To measure the QT interval for each heartbeat, we extracted the waveforms of individual heartbeats from continuous electrocardiogram data, treating each heartbeat as a distinct unit. Each heartbeat waveform extends from 120 ms before the R-wave peak to 80 ms before the peak of the subsequent R-wave. We standardized the duration of the heartbeat waveform to 1600 ms. The data exceeding this duration from the extraction point were truncated. Conversely, for heartbeat data of less than 1600 ms, we interpolated the end portions using the average of the two endpoints.

Subsequently, we excluded bradycardia, tachycardia, and heartbeat waveforms that exhibited considerable fluctuations in the RR interval. This exclusion was necessary because such data compromised the reliability of our correction formula and stability of the QT measurements. The specific criteria for exclusion were as follows: an RR interval from the preceding beat that was either less than 300 ms or more than 2000 ms, or an RR interval from the preceding beat that was less than 70 % or more than 130 % of the RR interval to the following beat.

Noise Removal: High-frequency noise was eliminated using a low-pass filter. The filter settings were configured with a passband edge at 50 Hz, a stopband edge at 60 Hz, a maximum passband loss of 3 dB, and a minimum stopband loss of 40 dB.

Data standardization: The potentials of the extracted heartbeat data were standardized to a mean of zero and a standard deviation of one. We calculated the standardized value z(ecg) of the extracted heartbeat data ecg using the following formula:

(1) $$\begin{array}{c} z\left(ecg\right)=\frac{ecg-\mu_{ecg}}{\sigma_{ecg}} \end{array}$$

where μ_ecg and σ_ecg represent the mean and standard deviation of the extracted heartbeat data, respectively.

### 2.2 Classification of the heartbeat data with K-shape clustering method

We categorized the extracted heartbeat data into clusters based on shape similarity using the K-shape method [31]. The number of clusters was set to 12. Clustering was performed using the KShape class from the Python TSlearn library (version 0.5.2). We configured both the number of initializations (n_init) and the maximum iterations for a single run (max_iter) to 100.

Clustering procedure.

#1 (Selecting initial centroids): We randomly selected 12 heartbeats from a set of heartbeats and assigned each to a different cluster. These are known as the initial centroids of the clusters.
#2 (Assigning heartbeats to the clusters): Each of the remaining heartbeats was assigned to the cluster corresponding to the initial centroid, which had the smallest Shape-Based Distance (SBD) to it. The precise definition of SBD between the two-time series is described later.
#3 (Generating new centroids): For each cluster, we calculated a new centroid by generating heartbeat data that minimized the total squared SBD from all heartbeats assigned to the cluster.
#4 (Reassigning heartbeats to the cluster): For each heartbeat, we calculated the SBDs from all centroids and then reassigned each heartbeat to the cluster whose centroid had the smallest SBD to that heartbeat.
#5 (Repeating #3 and #4): We iteratively repeated processes #3 and #4 to optimize the clustering. This was continued until the clusters no longer changed, or until the number of iterations reached 100, a limit we set as the “max_iter” parameter.

We repeated the aforementioned processes #1–5 a total of 100 times, which we set as the “n_init” parameter. During this repetition, the best clustering outcome was selected based on the criterion of minimal inertia. In this context, inertia is defined as the total squared SBD from each heartbeat to the centroid of its respective cluster. Clusters containing fewer than five heartbeats were excluded from the analysis.

The SBD between two time series $\vec{x}=\left(x_{1},・・・,x_{m}\right)$ and $\vec{y}=\left(y_{1},・・・,y_{m}\right)$ is defined as follows:

(2) $$\begin{array}{c} SBD\left(\;\vec{x},\vec{y}\right)=1-\max_{w}\;\left(\frac{{CC}_{w}\left(\;\vec{x},\vec{y}\right)}{\sqrt{R_{0}\left(\;\vec{x},\vec{x}\right)・\;R_{0}\left(\vec{y},\vec{y}\right)}}\right) \end{array}$$

where

(3) $$\begin{array}{c} {CC}_{w}\left(\;\vec{x},\vec{y}\right)={\;R}_{w-m}\left(\;\vec{x},\vec{y}\right),w\in \left\{1,2,\ldots 2m-1\right\} \end{array}$$

and

(4) $$\begin{array}{c} {\;R}_{k}\left(\vec{x},\vec{y}\right)=\left\{ \begin{array}{c} \sum_{l=1}^{m-k}x_{l+k}・\;y_{l},k\geq 0\\ {\;R}_{-k\;}\left(\vec{y},\vec{x}\right),k<0. \end{array}\right. \end{array}$$

### 2.3 Heartbeat data selection and template creation using DTW distance

We calculated the DTW distances from all the centroids to the heartbeats in their respective clusters based on the formula described in “Supplementary Methods.” Subsequently, for each cluster, the heartbeat with the smallest DTW distance to its centroid was selected as the template for the QT measurement of that cluster. We excluded heartbeats with DTW distances beyond a certain threshold (90th percentile or above) from the analysis.

A clinical laboratory technician with experience in interpreting electrocardiogram waveforms registered the positions of the starting point of the QRS wave and the end point of the T wave for each template. Registration of the positions is performed with the ECG enlarged to include approximately three beats per screen. After roughly aligning the positions, a clinical laboratory technician visually verifies and adjusts the registered positions in one-pixel increments to ensure they precisely align with the specified criteria. Once registration of the QRS onset and T-wave end for all 12 templates is complete, a simultaneous comparison of the templates is conducted to confirm consistent alignment across all templates, and corrections are made if needed.

### 2.4 Calculation of warping paths by applying the LDTW method

For each cluster, we determined the warping paths between the template and individual heartbeats using the LDTW method [32]. This method applies a restriction on the length of the warping path compared with that of the naive DTW method. The application of the DTW method to the ECG analysis was reported by Vulling et al. [33]. We also adopted the LDTW method instead of naive DTW to avoid anomalous warping paths, which could include a misalignment between the endpoints of the P and T waves. We used 'metrics.dtw_path_limited_warping_length' function of the Python TSlearn library to implement the LDTW method, with the ‘max_length’ parameter set to the length of the time series plus 10.

### 2.5 Calculation of QT Interval and Heartbeat Correction

Estimation of segmentation points in heartbeats: Using the registered positions in the template and the calculated warping paths, we estimated the start points of the QRS waves and the end points of the T waves of the heartbeats. In cases where multiple links existed for the starting point of the QRS wave or the end point of the T wave, we chose the latest point as the starting point of the QRS wave and the earliest point as the ending point of the T wave.

Calculation of the QT Interval and Heartbeat Correction: The QT intervals were directly calculated from the estimated start points of the QRS waves and the estimated endpoints of the T waves. Additionally, we applied the Bazett correction method to adjust the calculated QT intervals to QTcB time.

### 2.6 Evaluation of the validity of the developed algorithm

To evaluate the validity of the QT analysis method, we used data from the QT Database of PhysioNet, a public repository of two-channel ECG Holter recordings containing ECG data with diverse waveform patterns of the QRS and ST-T portions. Each ECG record in the QT database includes at least 30 annotated beats. Waveform boundaries were manually determined by expert annotators using an interactive graphic display, allowing simultaneous viewing of both signals for precise annotation. This database was specifically designed to facilitate the validation of ECG waveform analysis algorithms by encompassing a wide range of real-world ECG shapes [34,35].

It comprises 2-channel ECG data for 105 cases, with each case featuring a 15-min waveform with machine annotations. Additionally, it included expert-assigned PQRST coordinate annotations of 30–100 representative beats per case. First, we excluded cases that met the predefined technical exclusion criteria from the QT database. The details of the exclusion criteria are presented in Supplemental Table 1. Subsequently, we categorized the cases for analysis based on T-wave morphology. Specifically, the T-wave shapes were classified into five types: normal, negative, biphasic, flat, and high T-waves. The specifics of these categories are listed in Supplemental Table 2. This classification was performed manually by a clinical laboratory technician, and subsequently validated by a cardiology specialist.

We evaluated the performance of our QT analysis algorithm using ECG data classified by T-wave morphology. For this evaluation, we used physician-annotated data from the QT Database as the reference standard. Patients lacking annotated data were excluded. For each case, In the verification of the external data, analysis was conducted on heartbeats where experts had previously marked the QRS wave onset and T wave endpoint. The analysis sections consisted of 30–100 beats beginning 10 min after the start of each ECG recording, of which the first 15 beats were used for this evaluation.

For each T-wave shape, we calculated the mean and standard deviation of the QT and QTcB intervals using both our developed algorithm and physician annotations. To assess the concordance between our algorithm and the expert annotations, we computed the Intraclass Correlation Coefficient (2,1) for both the QT and QTcB intervals.

We also compared the developed algorithm with DSC5500 by Nihon Kohden, a commercially available Holter analysis software. In the system, the QT interval is calculated using the tangential method on 15-s segments of summation ECGs. For performance comparisons, we evaluated the differences in QT intervals and their standard deviations between the commercial software and our developed algorithm, using expert annotations as the reference. Performance was assessed for each T-wave morphology. To ensure consistency across the three QT interval calculation methods, we used the first 15 s of data with physician-annotated QT intervals from the QT Database for the evaluation.

We generated scatter plots and calculated the correlation coefficients to compare the QT intervals calculated using our algorithm with those derived from expert annotations. Additionally, we created Bland–Altman plots to evaluate the offset and proportional distortion. Finally, we assessed the clinical practicality of our algorithm by measuring the time required by a clinical technologist to perform QT analysis.

## 3 Results

### 3.1 Selection of appropriate ECG data for analysis from the QT database

To select waveforms to evaluate our algorithm, we reviewed the waveforms of 105 cases registered in the QT Database, adhering to the methods described in the Materials and Methods section. This included verifying annotation information and assessing waveform quality. After applying the predetermined technical exclusion criteria, 26 patients (24.8 %) were excluded. Consequently, 79 patients were included in the evaluation.

After preprocessing and clustering the heartbeat waveforms for the 79 cases selected for analysis, the total number of heartbeat waveforms analyzed was 71,905. Table 1 lists the number of cases and waveforms for each T-wave shape. We found that the T-wave shapes were normal in most cases, whereas a minority of cases exhibited either negative or biphasic T-wave morphologies.

**Table 1:** Number of cases to be analyzed for each T-wave shape.

	Number of cases	Number of heartbeat waveforms
Normal wave	56	51,062
Inverted T-wave	19	17,082
Biphasic T-wave	4	3761
All wave	79	71,905

Left column: Types of T-wave shapes.

Middle column: Number of cases.

Right column: Number of heartbeat data for each shape.

### 3.2 Implementation of QT analysis for each extracted heartbeat ECG data

Subsequently, for the 79 cases analyzed, we executed several steps: clustering heartbeat waveforms and creating templates using SBD, computing warping paths using the LDTW method, calculating QT intervals, and adjusting the heart rate to obtain corrected QT interval values (QTcB intervals). All 79 cases allowed for successful template registration and yielded QT and QTcB intervals. In the Supplemental Figure, we present examples of template registration and QT analysis results for normal, negative T, and biphasic T waveforms.

### 3.3 Evaluation of the QT and QTcB interval calculations and their consistency with expert manual assessments

**Fig. 1:**
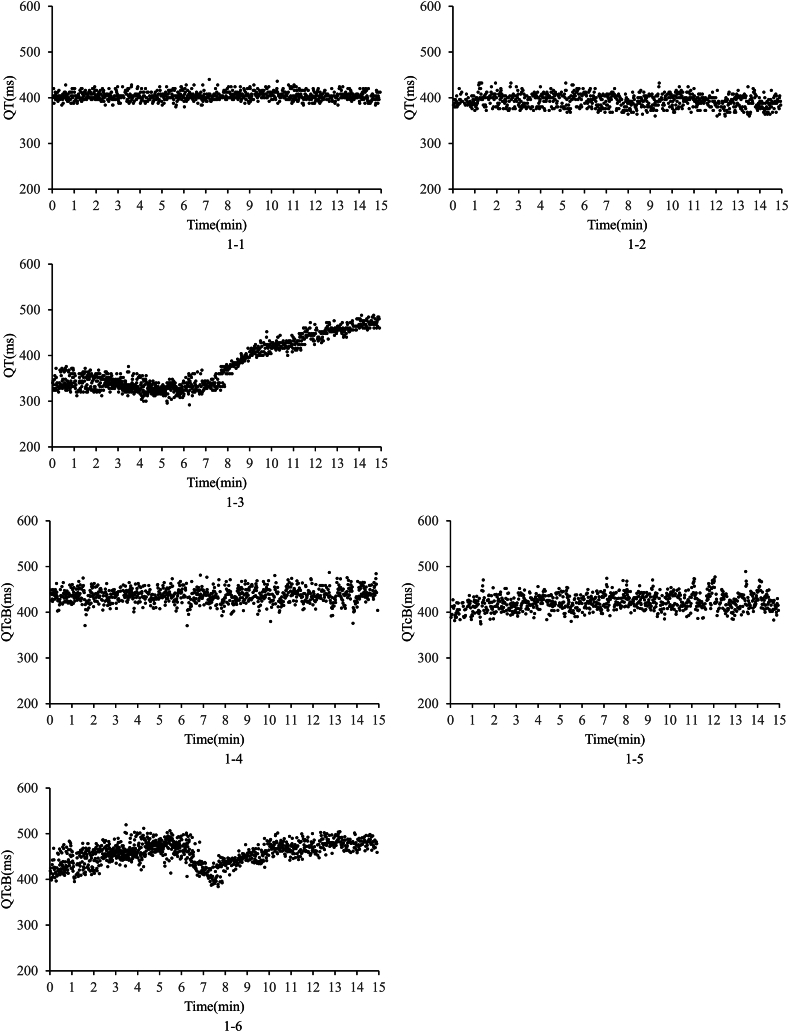
Time-series graph. Fig. 1–: Time-series graph of QT for 15 min of sel17453 (Normal wave). Figs. 1–2: Time-series graph of QT for 15 min of sel306 (Inverted T-wave). Figs. 1–3: Time-series graph of QT for 15 min of sel301 (Biphasic T-wave). Figs. 1–4: Time-series graph of QTcB for 15 min of sel17453 (Normal wave). Figs. 1–5: Time-series graph of QTcB for 15 min of sel306 (Inverted T-wave). Figs. 1–6: Time-series graph of QTcB for 15 min of sel301 (Biphasic T-wave).

Fig. 1 presents an example of time-series graph of the 15-min QT intervals calculated using our algorithm. Supplemental Table 3 presents QT and QTcB intervals calculated using the developed algorithm. In each table, we show the average values of the QT and QTcB intervals for 15-min, for each T-wave morphology and overall. Table 2, Table 3 present comparisons between the QT and QTcB intervals calculated using the developed algorithm and those derived from expert annotations. In each table, we show the average absolute values of the QT and QTcB intervals for all heartbeats, as well as for each T-wave morphology, along with the differences between them. In cases of normal or negative T wave shapes, the intervals calculated by our algorithm were generally longer than those from expert annotations, but these differences were typically within 1.0 ms for both QT and QTcB intervals.

**Table 2:** The QT intervals obtained from the developed algorithm and expert annotations.

	QT (novel QT analysis algorithm)	QT (specialist)	Difference
Normal wave	406.3 (12.0)	405.6 (14.9)	−0.8 (22.5)
Inverted T-wave	406.2 (12.5)	405.6 (15.7)	−0.6 (26.4)
Biphasic T-wave	443.5 (10.6)	443.6 (15.6)	0.1 (24.5)
All wave	408.2 (12.0)	407.5 (15.1)	−0.7 (23.6)

Mean (Standard Deviation), Unit: milliseconds.

**Table 3:** The QTcB intervals obtained from the developed algorithm and expert annotations.

	QTcB (novel QT analysis algorithm)	QTcB (specialist)	Difference
Normal wave	436.7 (15.9)	435.8 (17.6)	−0.9 (24.3)
Inverted T-wave	441.2 (14.5)	439.7 (18.3)	−1.5 (28.6)
Biphasic T-wave	481.8 (11.2)	483.0 (18.9)	1.2 (25.8)
All wave	440.1 (15.3)	439.1 (17.8)	−1.0 (25.5)

Mean (Standard Deviation), Unit: milliseconds.

Table 4 presents the results of concordance tests comparing the QT and QTcB intervals obtained using our developed algorithm with those derived from expert annotations. The Intraclass Correlation Coefficient (2,1) exceeded 0.7 across all categories, overall, in normal T wave, negative T wave, and biphasic T wave cases. These results indicate a high level of agreement between the QT/QTcB intervals calculated using our algorithm and those derived from expert annotations.

**Table 4:** ICC between the QT/QTcB interval obtained from the developed algorithm and the values obtained from expert annotations.

	QT		QTcB	
	ICC (2,1)	95 % CI	ICC (2,1)	95 % CI
All Wave	0.9	[0.89,0.91]	0.82	[0.8,0.84]
Normal wave	0.9	[0.89,0.91]	0.77	[0.74,0.8]
Inverted T-wave	0.89	[0.86,0.91]	0.82	[0.78,0.86]
Biphasic T-wave	0.82	[0.71,0.89]	0.94	[0.89,0.96]

The Intraclass Correlation Coefficient (2,1) for the QT interval calculated by the developed algorithm and expert human analysis was 0.95 [95 % CI: 0.92, 0.97], and it was 0.70 [95 % CI: 0.54, 0.81] for the QT interval calculated by the DSC 5500 and expert human analysis.

Table 5 presents the mean and standard deviation of the difference in QT intervals between the algorithms and the expert's analysis. For all waves, the mean and standard deviation of the difference between the algorithm and the expert analysis was 0.5 (±16), and that of the difference between the DSC5500 and the expert analysis was −16.4 (±42.8). For biphasic T-wave, the mean and standard deviation of the difference between the algorithm and the expert analysis was −0.3 (±15.9), and that of the difference between the DSC5500 and the expert analysis was −79.3 (±60.6).

**Table 5:** Difference analysis of QT interval measurements: Comparing machine methods versus manual specialist analysis.

	D from Specialist Measurements (msec)	
	Novel Algorithm	Commercial Software
Normal wave	1.3 (±13.8)	−6.8 (±35.7)
Inverted T-wave	−1.6 (±21.9)	−34.5 (±47.0)
Biphasic T-wave	−0.3 (±15.9)	−79.3 (±60.6)
All wave	0.5 (±16.0)	−16.4 (±42.8)

Values shown as: Difference (±Standard Deviation) in milliseconds.

D = Machine measurement - Specialist measurement.

The results showed that the developed algorithm performed better than the DSC5500 for all shapes, especially for biphasic T-wave.

**Fig. 2:**
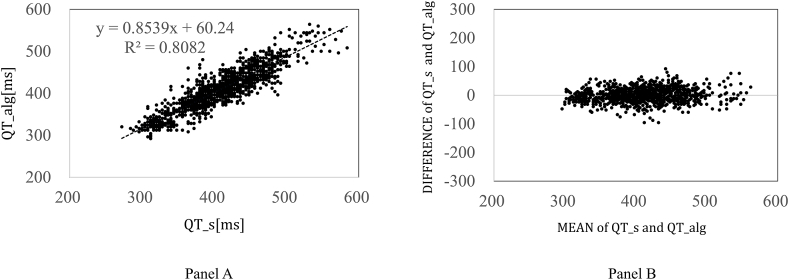
Scatter plot and Bland–Altman plot. Panel A: Scatter plot showing the correlation between the QT interval analyzed using the developed algorithm and the QT interval obtained from expert annotations. Unit: milliseconds. Panel B: Bland–Altman plot showing the correlation between the QT interval analyzed by the developed algorithm and the QT interval obtained from expert annotations. QT_s: The QT intervals obtained from the specialist. QT_alg: The QT intervals obtained from the novel QT analysis algorithm. Unit: milliseconds.

Fig. 2 shows scatter plots and Bland–Altman plots that illustrate the correlation between QT intervals calculated using our algorithm and those derived from expert annotations. The correlation coefficient (R) was 0.899, indicating a high degree of correlation. The Bland–Altman plots indicated minimal offset and proportional distortion, suggesting a low degree of error bias.

### 3.4 Evaluation of the work time required for QT analysis in the developed algorithm

The average time required by clinical laboratory technicians to analyze QT intervals for 15-min data using our developed algorithm was 13 min per case. The majority of this time was dedicated to template registration, whereas the remaining tasks were automated.

## 4 Discussion

In this study, we developed a new QT analysis algorithm for long-term ECGs by using shape-based clustering and a template-matching method with a limited search range. Using data from 79 cases in the QT database, we showed that the QT analysis algorithm can analyze the QT interval with high accuracy and that the analysis results are consistent with those obtained by physicians.

The first unique point of this study was to verify that the novel QT analysis algorithm can analyze the QT interval with high accuracy, even for waveforms with T-wave morphology changes. Specifically, we used 15 min of data from each of the 79 cases in the QT database to evaluate the overall T-wave shape classification.

Standard deviation of the time difference in the measured QT intervals was 23.6 ms between those evaluated by a specialist and those evaluated using the novel QT analysis algorithm. This value appears to be lower than that evaluated for T-wave terminals using the previous method (35.5 ms) [30]. Although the differences in the QT interval and T-wave endpoint were not identical, this suggests that the new QT analysis algorithm is highly accurate.

In addition, the results of the novel QT analysis algorithm demonstrated high concordance with the QT analysis results of specialists across various T-waveforms, including normal, negative, and biphasic. These findings suggest that the shape-based clustering method adopted in this study allows for high similarity between the ECG waveforms under analysis and the templates, implying an improvement in the accuracy of the QT interval analysis. Moreover, by using the LDTW method to limit the search range during alignment, the issues of abnormal alignment reported in the DTW method were mitigated, raising expectations for further improvements in the accuracy of QT interval analysis.

A second notable point of this study was the time efficiency achieved when performing QT interval analysis. As indicated by the results, the average time required for a technician to evaluate QT intervals was only 13 min per case. This facilitates the analysis of approximately 1000 heartbeats of long-term ECG data within a brief duration of work. Owing to the capability of the automatic classification of ECG waveforms into clusters of various unique shapes, an improvement in work time is anticipated as the length of the analyzed waveforms increases.

Specifically, the automatic extraction of template waveforms for each classified cluster eliminates the need to manually search for and match the correct data to ECG waveforms of diverse shapes over extended periods, such as 24 h. This factor is expected to reduce the work time and resources required for analysis compared with traditional methods [27,28], which require manual fitting to waveforms of various shapes. An additional advantage of this method is the reduction in the computational load achieved by limiting the number of templates for each heartbeat waveform to one, thereby enhancing the efficiency of computing resources.

Based on these factors, the template matching method using the newly developed automated template waveform creation method can accurately analyze QT intervals measured in long-term ECGs, regardless of T-wave morphological changes. This suggests the potential for the clinical application of this algorithm.

### 4.1 Limitation

In this analysis, among the 105 cases in the QT Database used for evaluation, 26 (25 %) were excluded based on pre-established exclusion criteria. The exclusion criteria included extreme bradycardia (HR < 30 bpm), arrhythmias (such as atrial flutter), low-voltage or noisy waveforms, inconsistent annotations, unqualified T-wave evaluations, and insufficient valuation of T waves.

Waveforms were excluded based on these criteria, whether due to the indeterminacy of the T-wave's end or their unsuitability for accurate QT interval measurement in cases of arrhythmia, for instance, highlighting a limitation. This suggests that a certain proportion of the waveforms present analytical challenges, which represents the first limitation of this study.

Another limitation is that as this study utilized an existing QT Database, it did not evaluate the variation in QT duration within the same case. Future assessments of the QT variations in the same case are expected to further enhance the reliability of the novel QT analysis algorithm proposed in this study.

### 4.2 Conclusion

In this study, a novel long-term ECG QT analysis algorithm was developed, utilizing shape-based clustering and template matching with a limited search range, and was evaluated using external data. The results confirmed that the algorithm could analyze QT intervals, which were highly consistent with the results obtained by physician analysis.

## CRediT authorship contribution statement

**Kaoru Hatano:** Writing – original draft, Visualization, Software, Methodology, Investigation, Funding acquisition, Formal analysis, Conceptualization. **Tomohiro Takata:** Writing – original draft, Validation, Software. **Mineki Takechi:** Validation, Supervision. **Akane Wada:** Investigation. **Hirohisa Taniguchi:** Investigation. **Yuichi Tamura:** Writing – review & editing, Writing – original draft, Visualization, Supervision, Conceptualization.

## Data availability statement

The data and codes were generated by Cardio Intelligence, Inc. (Tokyo, Japan). The data supporting the findings of this study are available from the corresponding author upon request.

## Funding

This research was financially supported by the PwC Foundation Grant Program 2021 and Grant Program for Starting Development of Medical Devices and Related Projects from the 10.13039/100019702Tokyo Metropolitan Government. Additionally, this study was conducted using research funds from Cardio Intelligence, Inc., with which the authors are affiliated.

## Declaration of competing interest

The authors declare the following financial interests/personal relationships which may be considered as potential competing interests: Yuichi Tamura reports financial support was provided by Cardio Intelligence Inc. Kaoru Hatano, Tomohiro Takata, Mineki Takechi, Akane Wada and Hirohisa Taniguchi reports was provided by Cardio Intelligence Inc. Yuichi Tamura has patent issued to assignee. Kaoru Hatano, Tomohiro Takata, Mineki Takechi, Akane Wada and Hirohisa Taniguchi has patent issued to assignee. Kaoru Hatano, Tomohiro Takata, Mineki Takechi, Hirohisa Taniguchi, and Yuichi Tamura are shareholders of Cardio Intelligence, Inc. If there are other authors, they declare that they have no known competing financial interests or personal relationships that could have appeared to influence the work reported in this paper.
