# Supplementary material for: Advanced QT interval analysis in long-term electrocardiography using shape-based clustering and template matching: A novel approach for Holter monitoring
Source: Heliyon. 2025 Feb 13;11(4):e42662. doi: 10.1016/j.heliyon.2025.e42662 (PMC11872472; doi:10.1016/j.heliyon.2025.e42662)
Supplement: Multimedia component 1 [file mmc1.docx]

Supplementary Material

# Supplementary Method

The calculation formula for Dynamic Time Warping (DTW) distance is as follows:

First, the alignment between two time series, $\vec{\boldsymbol{x}}\boldsymbol{=(}\boldsymbol{x}_{\boldsymbol{1}}\boldsymbol{,}\boldsymbol{・・・}\boldsymbol{,}\boldsymbol{x}_{\boldsymbol{m}}\boldsymbol{)}$ and $\vec{\boldsymbol{y}}\boldsymbol{=(}\boldsymbol{y}_{\boldsymbol{1}}\boldsymbol{,}\boldsymbol{・・・}\boldsymbol{,}\boldsymbol{y}_{\boldsymbol{m}}\boldsymbol{)}$, is represented by a set of n pairs, $\boldsymbol{W=}\left\{ \boldsymbol{w}_{\boldsymbol{1}}\boldsymbol{,}\boldsymbol{w}_{\boldsymbol{2}}\boldsymbol{,}\boldsymbol{・・・}\boldsymbol{,}\boldsymbol{w}_{\boldsymbol{n}} \right\}, with n\geq m$. Here, $\boldsymbol{w}_{\boldsymbol{k}}\boldsymbol{=}\left( \boldsymbol{i, j} \right)$**,**$\boldsymbol{1\leq i,j\leq m}$ indicates that the point at time i in time series $\vec{x}$ corresponds to the point at time j in time series $\vec{y}$. This sequence of correspondences $W$, which traces the path through both time series $\vec{x}$ and $\vec{y}$, is known as the warping path.

If we denote $\boldsymbol{w}_{\boldsymbol{k}}\boldsymbol{=}\left( \boldsymbol{w}_{\boldsymbol{k}}^{\boldsymbol{x}}\boldsymbol{,}\boldsymbol{w}_{\boldsymbol{k}}^{\boldsymbol{y}} \right)\boldsymbol{,1\leq k\leq n}$, then the set $W$ must satisfy the following conditions:

1. $\boldsymbol{w}_{\boldsymbol{k-1}}^{\boldsymbol{x}}\boldsymbol{-}\boldsymbol{w}_{\boldsymbol{k}}^{\boldsymbol{x}}\boldsymbol{\leq1 and}\boldsymbol{w}_{\boldsymbol{k-1}}^{\boldsymbol{y}}\boldsymbol{-}\boldsymbol{w}_{\boldsymbol{k}}^{\boldsymbol{y}}\boldsymbol{\leq1}$ (the path is continuous without interruption).

2. $\boldsymbol{w}_{\boldsymbol{k-1}}^{\boldsymbol{x}}\boldsymbol{-}\boldsymbol{w}_{\boldsymbol{k}}^{\boldsymbol{x}}\boldsymbol{\geq0 and}\boldsymbol{w}_{\boldsymbol{k-1}}^{\boldsymbol{y}}\boldsymbol{-}\boldsymbol{w}_{\boldsymbol{k}}^{\boldsymbol{y}}\boldsymbol{\geq0}$ (the path is monotonic without backtracking).

3. $\boldsymbol{w}_{\boldsymbol{1}}\boldsymbol{=}\left( \boldsymbol{1,1} \right)\boldsymbol{and}\boldsymbol{w}_{\boldsymbol{n}}\boldsymbol{=(m,m)}$ (the starting point of the path is (1,1) and the end point is (m,m)).

For the warping path W, the cost function $cost(W)$is defined as follows:

$$\begin{aligned} \boldsymbol{cost}\left( \boldsymbol{W} \right)\boldsymbol{=}\sqrt{\sum_{\mathbf{k=1}}^{\mathbf{n}} \left| \boldsymbol{x}_{\boldsymbol{w}_{\boldsymbol{k}}^{\boldsymbol{x}}}\boldsymbol{-}\boldsymbol{y}_{\boldsymbol{w}_{\boldsymbol{k}}^{\boldsymbol{y}}} \right|^{\boldsymbol{2}}}\boldsymbol{\#}\left( \boldsymbol{5} \right) \end{aligned}$$

The cost function acts as a measure of the alignment quality. Considering all possible warping paths that meet conditions 1-3, the minimum value of $cost\left( W \right)$ defines the DTW distance between time series $\vec{x}$ and$\vec{y}$. and can be expressed as follows:

$$\begin{aligned} \boldsymbol{DTW}\left( \vec{\boldsymbol{x}}\boldsymbol{,}\vec{\boldsymbol{y}} \right)\mathbf{=}\min_{\boldsymbol{W}} \sqrt{\sum_{\mathbf{k=1}}^{\mathbf{n}} \left| \boldsymbol{x}_{\boldsymbol{w}_{\boldsymbol{k}}^{\boldsymbol{x}}}\boldsymbol{-}\boldsymbol{y}_{\boldsymbol{w}_{\boldsymbol{k}}^{\boldsymbol{y}}} \right|^{\boldsymbol{2}}}\boldsymbol{\#}\left( \boldsymbol{6} \right) \end{aligned}$$

# Supplementary Tables

Supplemental Table 1: Details of the exclusion criteria

| Exclusion criteria | Details of the Exclusion criteria | Number of cases |
| --- | --- | --- |
| 1.　Factors of Cardiac Rhythm | 1-1. Extreme Bradycardia  (HR<30 bpm): | 1 |
|  | 1-2. Arrythmia  (Atrial Flutter, Atrial fibrillation) | 2 |
| 2.　Technical Error | 2-1. Less valuation of T waves | 1 |
| 3. Factors of Record | 3-1. Low Voltage | 3 |
|  | 3-2. Noisy Waveforms | 6 |
| 4. Inappropriate Samples | 4-1. Inconsistent Annotations | 3 |
|  | 4-2. Unqualified T-wave evaluations | 10 |
| Total | | 26 cases |

Supplemental Table 2: Details of the classification of T-wave shapes

|  | Classification criteria |
| --- | --- |
| Normal wave | The height of the T-wave is 1.5 times that of the P-wave, and between 1/2 to 1/8 of the R-wave  The shape of the T-wave is typically upward in the first two-thirds and downward in the remaining one-third |
| Inverted T-wave | A downward T-wave relative to the baseline |
| Flat T-wave | The height of the T-wave is less than 1/10 of the height of the R-wave |
| Biphasic T-wave | The initial part of the T-wave is negative, and the ending part is positive, or the initial part is positive, and the ending part is negative |
| High T-wave | The height of the T-wave is more than 1.0 mV above the baseline |

Supplemental Table 3: The QT/QTcB intervals obtained from the developed algorithm for 15min data

|  | QT (novel QT analysis algorithm) | QTcB (novel QT analysis algorithm) |
| --- | --- | --- |
| Normal wave | 404.9 (15.0) | 437.4 (18.7) |
| Inverted T-wave | 405.1 (15.5) | 438.9 (19.8) |
| Biphasic T-wave | 430.7 (22.1) | 482.1 (19.5) |
| All wave | 406.3 (15.5) | 440.0 (19.0) |

Mean (Standard Deviation), Unit: milliseconds

## Supplementary Figures


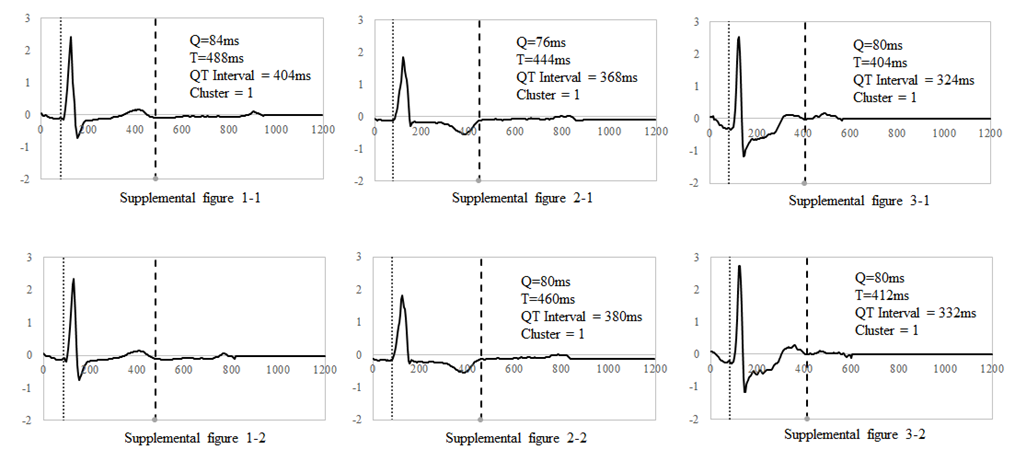


**Supplementary Figure 1.** Examples of template registration and QT analysis result. Supplemental figure 1-1: Template for sel17453 (Normal wave)

Supplemental figure 1-2: QT analysis result of sel17453 (Normal wave)

Supplemental figure 2-1: Template for sel306 (Inverted T-wave)

Supplemental figure 2-2: QT analysis result of sel306 (Inverted T-wave)

Supplemental figure 3-1: Template for sel301 (Biphasic T-wave)

Supplemental figure 3-2: QT analysis result of sel301 (Biphasic T-wave)

The dotted line indicates the start point of the QRS wave and the dashed line indicates the end point of the T wave. The values in the upper-right corner of each graph indicate the coordinates at the start of the QRS wave, at the end of the T wave, the QT interval, and the cluster to which it belongs. It can be seen that in all four cases, the Q and T coordinates were successfully identified.
